# Microbial quality and formation of biogenic amines in the meat and edible offal of *Camelus dromedaries* with a protection trial using gingerol and nisin

**DOI:** 10.1002/fsn3.1503

**Published:** 2020-03-12

**Authors:** Hui Tang, Wageh Sobhy Darwish, Waleed Rizk El‐Ghareeb, Naser A. Al‐Humam, Lin Chen, Rui‐Min Zhong, Zi‐Jun Xiao, Jin‐Kui Ma

**Affiliations:** ^1^ Henry Fok School of Food Science and Engineering Shaoguan University Shaoguan China; ^2^ Food Control Department Faculty of Veterinary Medicine Zagazig University Zagazig Egypt; ^3^ Department of Health Science and Technology Faculty of Health Sciences Hokkaido University Sapporo Japan; ^4^ Department of Veterinary Public Health and Animal Husbandry College of Veterinary Medicine King Faisal University Hofuf Saudi Arabia; ^5^ Department of Microbiology and Parasitology College of Veterinary Medicine King Faisal University Hofuf Saudi Arabia; ^6^ School of Food & Pharmaceutical Engineering Zhaoqing University Zhaoqing China

**Keywords:** camel meat, gingerol, microbial quality, nisin, offal

## Abstract

This study aimed firstly at the investigation of the microbial status of the camel meat and edible offal including liver, kidneys, lungs, rumen, and duodenum distributed at local markets of Egypt. Total plate count, total psychrophilic counts, total Enterobacteriaceae count, the most probable number of coliforms, and total mold counts were monitored at the collected samples. The produced biogenic amines (BA) in the camel meat and offal were further estimated. An experimental trial to investigate the antimicrobial potentials of either nisin, gingerol, or an equal mixture of both using camel muscle as a food matrix was conducted. The achieved results declared a high microbial load in the camel meat and the offal. Duodenum and rumen had the highest microbial counts followed by lungs, kidneys, liver, and muscle, respectively. Similarly, duodenum and rumen had the highest levels of BA, including tyramine, spermine, putrescine, cadaverine, and histamine. Both of nisin and ginger showed significant antimicrobial properties in a concentration‐dependent manner. Thus, efficient hygienic measures during the handling of camel meat are highly recommended. In addition, using nisin, gingerol, or a mixture of both is an efficient strategy for improving the microbiological quality of the camel meat.

## Graphical abstract

This study aimed firstly at investigation of the microbial status of the camel meat and edible offal including liver, kidneys, lungs, rumen and duodenum distributed at local markets of Egypt. The achieved results declared high microbial load in the camel meat and the offal. Both of nisin and ginger showed significant antimicrobial properties in a concentration‐dependent manner. Thus, efficient hygienic measures during handling of camel meat are highly recommended. Adoption of strict hygienic measure and using nisin and gingerol are recommended.

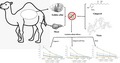

## 1 INTRODUCTION

Camel (*Camelus dromedaries*) meat and offal are considered as major sources for animal‐derived protein in many parts of the world. These kinds of meat are rich in protein, polyunsaturated fatty acids, and minerals. Camel meat is considered healthier than that of beef and mutton, in terms of less amount of fat, less cholesterol with high protein content (Kadim, Mahgoub, & Purchas, 2008). Camel meat is believed to have many medical values for the treatment of diseases in several Arabian countries, India, and Australia. Camel meat production is increasing worldwide and currently represents about 0.7% of the total world meat production (Suliman, Fadlalmola, Babiker, Arabi, & Ibrahim, 2014).

The high content of protein and moisture are considered as ideal factors that enhance microbial spoilage of the meat and offal. In addition, poor hygienic measures adopted during slaughtering, evisceration, distribution, and storage of the meat and offal are additional factors for the rapid onset of the microbial spoilage of the meat (Darwish, Eldin, & Eldesoky, 2015). However, there is a clear lack of information about the microbial quality of camel meat and offal in the local markets of Egypt. Indicators of the microbial quality and hygienic status of meat include total mesophilic (plate) count (TPC), total psychrophilic counts (TPsC), total *Enterobacteriaceae* count (TEC), the most probable number of coliforms (MPN), and total mold counts (TMC) (Vanderzant & Splittstroesser, 2001). One major task of the food control sector is to ensure the safety of marketed meat, and consumer protection. Therefore, the microbial quality of the marketed camel meat and offal in Egypt was investigated in the present work.

Biogenic amines (BA) are nitrogenous compounds that are formed through amino acid decarboxylase reactions. Biogenic amines may be formed through autolysis or proteolysis by proteolytic microorganisms (Ruiz‐Capillas & Jimenez‐Colmenero, 2005). Biogenic amines are formed in different food matrices such as meat, fish, milk, and vegetables. Consumption of biogenic amines‐contaminated foods may lead to several toxicological implications such as anaphylaxis, nervous, and muscular disorders (Stadnik & Dolatowski, 2010). Concentrations of BA in the food give an indication about the microbial quality of the food and the hygienic status adopted during preparation and processing. However, there is a clear lack of information about the contents of BA in camel meat and offal.

Gingerol is one of the active compounds found in ginger with several biological activities. Of these, its antioxidant and antimicrobial effects as reported before are against periodontal bacteria (Park, Bae, & Lee, 2008). The mechanisms behind gingerol‐antibacterial activities are still unclear. In addition, the ameliorative effects of gingerol on the microbial status and formation of BA in the meat had scarcely investigated.

Nisin is an antibacterial peptide that is secreted by several strains of *Lactococcus lactis*, it has a well‐documented bacterial inhibitory effect against a vast array of microorganisms (Abts et al., 2011). The mechanism of the inhibitory effects of nisin is mainly via binding to lipid II, an essential cell wall precursor, thus prevents cell wall synthesis (Hasper, de Kruijff, & Breukink, 2004). However, the preservative effects of nisin on the camel meat had received less attention.

Insight of these factors, the objectives of this study were first to investigate the microbial status of the camel meat and offal (liver, kidney, lungs, rumen, and duodenum) collected from different localities in Egypt via estimation of TPC, TPsC, MPN, TEC, and TMC. Levels of BA in the camel meat and offal were quantitatively estimated using the amino acid analyzer. An experimental trial to investigate the ameliorative effects of either nisin, gingerol, or an equal mixture of both on the microbial quality of the camel meat was conducted.

## 2 MATERIALS AND METHODS

The experimental work in the present study is divided into two experiments. Experiment 1 aimed at the investigation of the microbial status and BA content in the camel meat and edible offal. Experiment 2 aimed at investigating the antimicrobial potentials of either nisin, gingerol or an equal mixture of both using camel muscle as a food matrix.

### 2.1 Experiment 1

#### 2.1.1 Collection of samples

One hundred and twenty samples including 20 each of camel meat (round), liver, kidneys, lungs, rumen, and duodenum were collected randomly from different butchery shops in Egypt. Samples were collected from October 2017 to March 2018. The collected samples were transferred cooled directly without delay to the laboratory for the microbiological examination.

#### 2.1.2 Microbiological examinations

Samples were prepared according to recommendations of the American Public Health Association (APHA) (Vanderzant & Splittstroesser, 2001). In brief, 25 g of each sample weighed and homogenized with 225 ml of 0.1% of sterile buffered peptone water (LAB104, LAB M) for 1–2 min at 450 *g* using a sterile homogenizer (type M‐p3‐302, mechanic, precyzina). Such homogenate represents the dilution of 10^−1^ and then followed by the preparation of tenfold decimal serial dilutions.

#### 2.1.3 Total plate count (TPC)

Total plate count was estimated using the method of APHA (Vanderzant & Splittstroesser, 2001). In brief, one ml from each dilution was pipetted to a clean and sterile Petri dish. About 12–15 ml of plate count agar (Difco Laboratories) cooled to 45 ± 1°C were added to each Petri dish and left to solidify at room temperature and then incubated at 35 ± 2°C for 48 hr. Colonies including pinpoint size were counted as TPC in plates with 25–250 colonies per dish.

TPC/g=average No. of colonies×reciprocal of the dilution

#### 2.1.4 Determination of total psychrophilic count (TPsC)

The same method used for TPC was performed. Petri dishes were incubated at 7°C for 10 days. Results were calculated and recorded in the same way as TPC.

#### 2.1.5 Determination of the most probable number (MPN) of Coliforms

Three tubes’ most probable number (MPN) method was used (Vanderzant & Splittstroesser, 2001). In brief, one ml of each dilution was used to inoculate separately into three test tubes containing MacConkey broth with inverted Durham's tubes. The inoculated tubes were incubated at 37°C for 24–48 hr. Positive tubes showing acid (yellow color) and gas production in inverted Durham's tubes were recorded. The most probable number of coliforms was calculated according to the recommended tables.

#### 2.1.6 Determination of total Enterobacteriaceae count (TEC)

From the original and the subsequently prepared dilutions, one ml was poured onto an empty presterilized Petri dish, and then 12–15 ml of violet red bile glucose agar (Difco Laboratories) cooled to 45 ± 1°C was poured to each Petri dish. The plates were incubated at 37°C for 24 hr. All large pink to red colonies were counted (Vanderzant & Splittstroesser, 2001). Results were calculated and recorded in the same way as TPC.

#### 2.1.7 Determination of the total mold count (TMC)

Total mold counts were determined by culturing duplicate plates on Sabouraud's dextrose agar media (Oxoid) supplemented with chloramphenicol 100 mg/L followed by incubation in dark at 25°C for 5–7 days. During the incubation time, the plates were examined daily for fungal growth. Estimation of total mold was obtained by direct counting of the cultured agar plates (Vanderzant & Splittstroesser, 2001).

TMC/g=average No. of colonies×reciprocal of the dilution

#### 2.1.8 Content of biogenic amines (BA)

The extraction of BA was conducted by homogenizing 10 g of each sample with 100 ml of 10% trichloroacetic acid. The homogenate had been extracted for 1 hr followed by centrifugation at 2,800 *g*, 4°C for 20 min. The supernatants were filtered through a Whatman filter No. 1. The filtrates were stored at 4°C until analysis. The analysis of biogenic amines was performed using an amino acid analyzer (L‐8900, HITACHI), as described in a recent study (Kononiuk & Karwowska, 2019). Content of the BA (histamine, tyramine, putrescine, cadaverine, spermidine, agmatine, and spermine) was determined with a reference to the amine standards (Merck KGaA). Detection limits for the tested BA ranged from 0.004 to 0.01 mg/g of meat. The biogenic amine concentrations were reported as mg/g meat.

### 2.2 Experiment 2

#### 2.2.1 Improvement of the microbiological status of camel meat using gingerol and nisin

Reduction trials for the microbial load of camel meat were done using food grades of 6‐gingerol (Biopurify Phytochemicals) and nisin (Food grade, SIDLEY chemical, Linyi city, China) under 0.5, 1.5, and 2.5% separate or in a combination. A block of camel meat (5 kg) was purchased from the local market. This block was divided into 10 groups (each group includes five meat pieces, 100 g/piece) and used in the experimental trial. The 1st group was soaked in corn oil for 30 min at room temperature and used as a control (nontreated) in this study. The 2nd, 3rd, and 4th groups were soaked for 30 min at room temperature in nisin 0.5%, 1.5%, and 2.5% in corn oil, respectively. The 5th, 6th, and 7th groups were soaked in 6‐gingerol 0.5%, 1.5%, and 2.5% in corn oil, respectively at the same conditions. The synergistic effects of equal volumes of nisin and 6‐gingerol were additionally tested. The 8th, 9th, and 10th groups were soaked for 30 min at room temperature in nisin/gingerol (1:1) 0.5%, 1.5%, and 2.5% in corn oil, respectively. TPC, TPsC, MPN, TEC, TMC, and changes in the content of the BA were evaluated as mentioned before.

#### 2.2.2 Statistical analysis

All values were expressed as means ± *SE*, and all measurements were carried out in duplicates. Bacterial counts were converted into base logarithms of colony‐forming units per g (log CFU/g). Statistical significance was evaluated using the Tukey–Kramer HSD test. In all analyses, *p* < .05 was taken to indicate statistical significance using JMP statistical package; SAS Institute Inc.

## 3 RESULTS AND DISCUSSION

### 3.1 Experiment 1

#### 3.1.1 Microbial status of camel meat and edible offal

**Figure 1:**
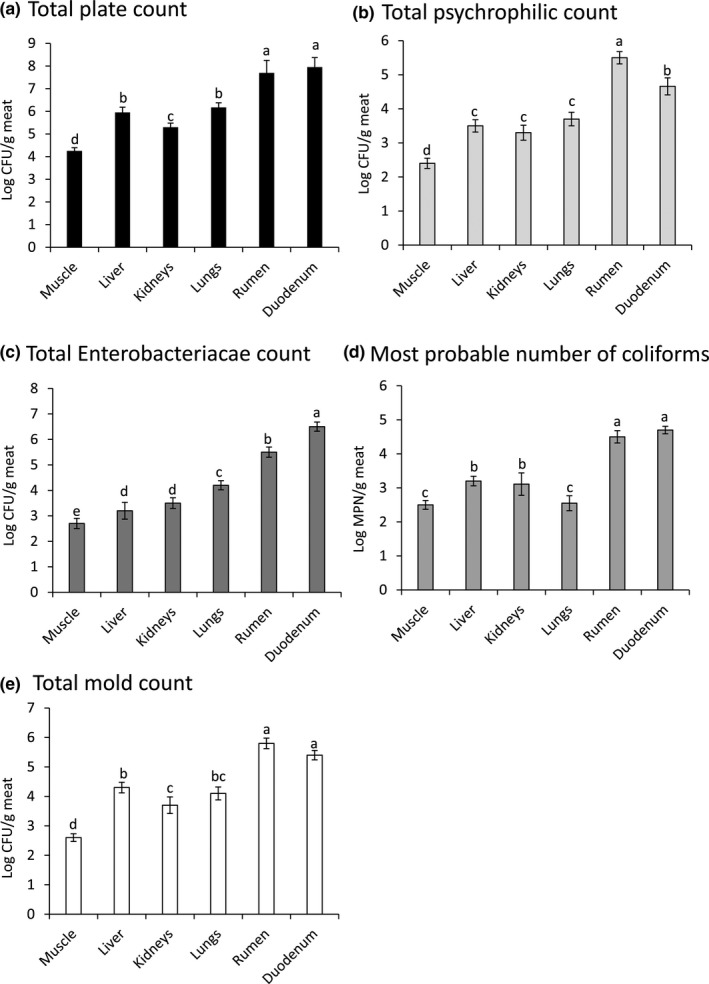
Hygienic status of camel meat and offal. (a) Total plate count, (b) total psychrophilic count, (c) total Enterobacteriaceae count, (d) most probable number of coliforms, and (e) total mold count of the examined camel meat and edible offal including duodenum, rumen, lungs, kidneys, and liver. Values represent means ± *SE* (Log CFU/g) of twenty samples from each. Columns carrying different superscript letters differ significantly among examined samples at *p* < .05

The microbial status of the examined camel meat and edible offal samples was investigated using TPC, TEC, MPN, TPsC, and TMC. The recorded results indicated that duodenum and rumen had significantly (*p* < .05) the highest TPC followed by lungs, liver, kidneys, and finally muscle. The mean values of the total plate counts were 7.95 ± 0.42, 7.69 ± 0.55, 6.18 ± 0.21, 5.95 ± 0.23, 5.30 ± 0.18, and 4.26 ± 0.14 log CFU/g in the examined duodenum, rumen, lungs, liver, kidneys, and muscle, respectively (Figure 1a). Rumen had significantly the highest total psychrophilic count followed by the duodenum. The recorded mean counts were 5.50 ± 0.18, 4.66 ± 0.25, 3.70 ± 0.20, 3.50 ± 0.18, 3.30 ± 0.22, and 2.40 ± 0.15 log CFU/g in the examined rumen, duodenum, lungs, liver, kidneys, and muscle, respectively (Figure 1b). The achieved results in this study go in agreement with other reports which indicated high total bacterial counts in fresh camel meat and beef marketed in Iran and Egypt (El‐Wehedy, Darwish, Tharwat, & Hafez, 2019; Fallah, Tajik, Razavi Rohani, & Rahnama, 2008). The total microbial counts give an indication about the sanitary status and recontamination of meat and meat products (Reij, Den Aantrekker, & ILSI‐Europe Risk Analysis in Microbiology Task Force., 2004). The high total bacterial count in this study indicates the poor hygienic conditions adopted during handling, storage, and processing of the camel meat. It might also indicate cross‐contamination of the meat from the workers’ hands, cutting boards, water, and knives used during slaughtering, evisceration and processing of the camel meat and offal. Coliform bacteria are considered as significant microbiological sanitary indicators, which emphasizes hygiene in all steps of preparation and handling of food subjects (Darwish et al., 2015). The recorded mean values of TEC were 6.50 ± 0.18, 5.50 ± 0.20, 4.20 ± 0.18, 3.50 ± 0.21, 3.20 ± 0.33, and 2.70 ± 0.20 log CFU/g in the examined duodenum, rumen, lungs, kidneys, liver, and muscle, respectively (Figure 1c). Most probable number of coliforms (Log MPN/g tissue) had relatively the same trend, as duodenum had the highest MPN value (4.70 ± 0.11) followed by rumen (4.50 ± 0.18), kidneys (3.11 ± 0.33), liver (3.20 ± 0.14), lungs (2.55 ± 0.22), and muscle (2.50 ± 0.13), respectively (Figure 1d). In agreement with the results of this study, Rahimi et al. isolated fecal coliforms including Shiga‐toxin‐producing *E. coli* from raw beef, camel, sheep, goat, and water buffalo meat in Fars and Khuzestan provinces, Iran (Rahimi, Kazemeini, & Salajegheh, 2012). Furthermore, El‐Wehedy et al. used TEC as an indicator for the hygienic status of raw meat served at hospitals in Egypt and they recorded unsatisfactory hygienic measures (El‐Wehedy et al., 2019). However, TEC was below the detection limit in camel carcasses slaughtered at Sahrawi refugee camps, Algeria (Corrò et al., 2012). The coliform counts are suggested to be used as a broad base indicating fecal contamination of meat and meat products due to inadequate processing and postprocessing recontamination (International Commission of Microbiological Specification for Foods (ICMSF), 1996).

Total mold counts were also estimated in the present study, and the recorded TMC were 5.50 ± 0.18, 4.66 ± 0.25, 3.70 ± 0.20, 3.50 ± 0.18, 3.30 ± 0.22, and 2.40 ± 0.15 log CFU/g in the examined rumen, duodenum, lungs, liver, kidneys, and muscle, respectively (Figure 1e). In agreement with the results of this study, Fallah et al. recorded high mold counts in the refrigerated camel meat collected from different markets in Iran (Fallah et al., 2008). Fungal contamination of meat in this study indicates inadequate sanitary measures performed starting from slaughtering, evisceration, and storage. The conditions of the environment in the refrigerators, cutting boards and workers’ hands, and clothes are very suitable for the development of mold spores (Mižáková, Pipová, & Turek, 2002; Reij, Den Aantrekker, & ILSI‐Europe Risk Analysis in Microbiology Task Force., 2004). Fungal contamination of meat may lead to their spoilage and production of mycotoxins with potential health hazards to humans due to their carcinogenic effects, liver diseases, and organ damage (Darwish, Ikenaka, Nakayama, & Ishizuka, 2014).

The overall obtained results in the current investigation revealed the lack of proper hygienic measures adopted during the handling and marketing of the examined camel meat and edible offal, leading to poor microbial quality of such products. Edible offal had significantly higher microbial load compared with the meat. This could be explained as offal usually receives less attention during washing or preparation compared with the muscle and may be kept in the wastewater until the time of marketing (Darwish et al., 2018).

**Figure 2:**
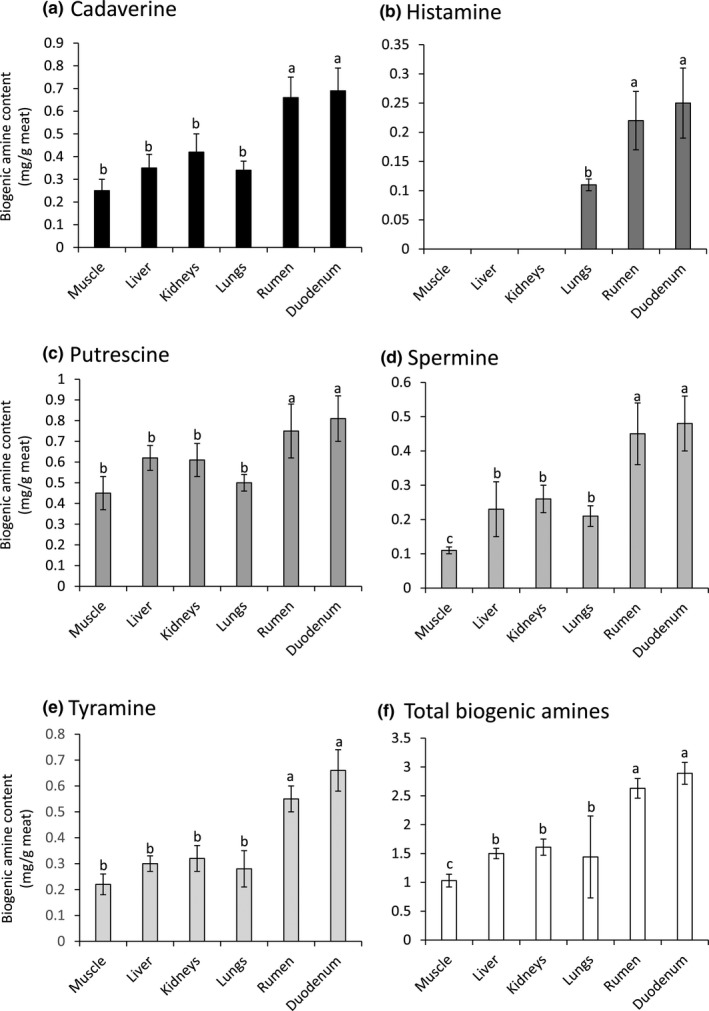
Biogenic amine content in the camel meat and offal. The content of (a) cadaverine, (b) histamine, (c) putrescine, (d) spermine, and (e) tyramine, and total biogenic amines in the examined camel meat and edible offal including duodenum, rumen, lungs, kidneys, and liver. Values represent means ± *SE* (mg/g) of twenty samples from each. Columns carrying different superscript letters differ significantly among examined samples at *p* < .05

Production of BA gives an indication about the shelf life and the hygienic status of the meat and meat products (Ruiz‐Capillas & Jimenez‐Colmenero, 2005). The recorded levels of BA in the current study revealed that duodenum followed by rumen had the highest BA concentrations. The recorded total BA levels were 2.89 ± 0.19, 2.63 ± 0.17, 1.61 ± 0.14, 1.50 ± 0.09, 1.44 ± 0.71, and 1.03 ± 0.11 mg/g in the examined duodenum, rumen, kidneys, liver, lungs, and muscle, respectively (Figure 2). The recorded concentrations of BA in the examined samples go in line with the hygienic status of the tested samples. To the best of our knowledge, there is no information about the content of BA in the edible offal. The estimated BA content in the muscle goes in context with that reported by Kukleci et al, who recorded elevated concentrations of BA associated with high levels for the hygiene indicator bacteria in ready‐to‐eat meat products at retail in the Republic of Kosovo (Kukleci, Smulders, Hamidi, Bauer, & Paulsen, 2019). Five biogenic amines were detected, and putrescine recorded the highest levels detected at the examined tissues followed by cadaverine > tyramine > spermine > histamine (Figure 2). Likely, Balamatsia et al. confirmed the possible role of BA as spoilage indicators, particularly, putrescine, cadaverine, and tyramine (Balamatsia, Paleologos, Kontominas, & Savvaidis, 2006). In addition, Jastrzębska et al. demonstrated that putrescine and cadaverine were the highest BA detected in meat samples collected from Poland (Jastrzębska, Kowalska, & Szłyk, 2016).

### 3.2 Experiment 2

#### 3.2.1 Improvement of the microbial status of the camel meat using nisin and gingerol

Microbial contamination of meat had several implications starting from organoleptic changes such as off‐flavor and softening of the muscular tissue leading to a reduction in the keeping quality of the final product and subsequently massive economic losses due to the poor marketing of such products, and the most serious implication is related to food‐poisoning if such contaminated product is consumed. Thus, one major task of the food hygienists and microbiologists is to find ways to extend the shelf life of meat and to decontaminate or improve the microbial quality of meat and meat products. Thus, in the second part of this study, trials to reduce the microbial contamination of the camel meat were conducted using nisin, gingerol, or an equal mixture of both.

The achieved results in Table 1 indicated that nisin could significantly reduce the bio‐indicator parameters in the camel meat in a concentration‐dependent manner. For instance, nisin 2.5% significantly reduced TPC, TPsC, TEC, MPN, and TMC at 58.35%, 49.18%, 59.56%, 50.59%, and 53.01%, respectively. Similarly, nisin reduced the formed BA either individually or in total in a concentration‐dependent manner (Table 2). In agreement with the obtained results, Avery and Buncic recorded the anti‐Listerial activity of nisin especially in an in vitro approach (Avery & Buncic, 1997). Furthermore, He et al. confirmed the preservative effects of nisin against Gram‐positive organisms using the agar dilution method (He et al., 2016). On line with the antimicrobial properties of nisin, gingerol 1.5% significantly (*p* < .05) reduced TPC (47.76%), TPsC (40.57%), TEC (47.06%), MPN (38.25%), and TMC (29.32%). Such reduction percentages were increased to 63.53%, 50.00%, 63.24%, 55.78%, and 57.14% respectively, after immersion in the gingerol 2.5% for 30 min (Table 1). Interestingly, gingerol reduced the formed BA in a dose‐response manner as indicated in Table 2. In correspondence with the achieved results in the present study, Cao et al. used aqueous extract of ginger at 5% and 10% to improve the quality and shelf life of stewed‐pork during refrigerated storage (Cao et al., 2013). Furthermore, Abdel‐Naeem and Mohamed used the ginger extract to improve the sensory and the physicochemical properties of the camel burger patties in Egypt (Abdel‐Naeem & Mohamed, 2016). There is a clear lack of information about the synergistic antimicrobial action of both nisin and gingerol. Thus, the synergistic antimicrobial effects of a combination of both nisin and gingerol (1:1) at different concentrations were tested. The obtained results indicated that nisin–gingerol extracts significantly reduced TPC, TPsC, TEC, MPN, and TMC in a concentration‐dependent manner. The reduction percentages of nisin–gingerol 0.5% for these parameters were 32.24%, 33.61%, 26.47%, 25.09%, and 24.81%, respectively. However, at 2.5%, the reduction levels were significantly increased to 64.71%, 59.02%, 63.24%, 60.16%, and 62.41%, respectively (Table 1). Furthermore, the used nisin–gingerol combination significantly reduced the formed BA in a concentration‐dependent fashion (Table 2). Similarly, Cao et al. had successfully used ginger at a combination with onion and garlic to improve the shelf life of stewed‐pork during refrigerated storage (Cao et al., 2013). Furthermore, Gharsallaoui et al. mentioned that nisin has strong antimicrobial abilities and can be used alone or in combination to improve the microbial quality of the meat and meat products (Gharsallaoui, Oulahal, Joly, & Degraeve, 2016). Therefore, a combination of nisin–gingerol 2.5% is highly recommended to achieve the highest improvement in the microbial quality of the camel meat.

**Table 1:** Antimicrobial activities of nisin, gingerol, and a combination of both at different concentrations in camel meat

	TPC		TPsC		TEC		MPN		TMC	
	Count (Log cfu/g)	Reduction (%)	Count (Log cfu/g)	Reduction (%)	Count (Log cfu/g)	Reduction (%)	Count (Log MPN/g)	Reduction (%)	Count (Log cfu/g)	Reduction (%)
Control	4.25 ± 0.14^a^	0	2.44 ± 0.15^a^	0	2.72 ± 0.21^a^	0	2.51 ± 0.13^a^	0	2.66 ± 0.13^a^	0
Nisin 0.5%	3.11 ± 0.18^b^	26.82	2.00 ± 0.11^ab^	18.03	2.24 ± 0.14^b^	17.65	2.21 ± 0.15^ab^	11.95	2.11 ± 0.24^b^	20.68
Nisin 1.5%	2.54 ± 0.12^c^	40.24	1.65 ± 0.24^bc^	32.38	1.66 ± 0.18^cd^	38.97	1.77 ± 0.16^bc^	29.48	1.77 ± 0.11b^b^	33.46
Nisin 2.5%	1.77 ± 0.11^de^	58.35	1.24 ± 0.14^cd^	49.18	1.10 ± 0.11^de^	59.56	1.24 ± 0.14^cde^	50.59	1.25 ± 0.16^cd^	53.01
Gingerol 0.5%	3.00 ± 0.15^b^	29.41	1.80 ± 0.21^b^	26.23	2.08 ± 0.09^bc^	23.53	2.00 ± 0.18^b^	20.32	2.00 ± 0.15^b^	24.81
Gingerol 1.5%	2.22 ± 0.15^cd^	47.76	1.45 ± 0.14^bc^	40.57	1.44 ± 0.11^d^	47.06	1.55 ± 0.17^cd^	38.25	1.88 ± 0.12^bc^	29.32
Gingerol 2.5%	1.55 ± 0.17^e^	63.53	1.22 ± 0.12^cd^	50	1.00 ± 0.12^e^	63.24	1.11 ± 0.12^de^	55.78	1.14 ± 0.11^d^	57.14
Nisin + Gingerol 0.5%	2.88 ± 0.16^bc^	32.24	1.62 ± 0.10^b^	33.61	2.00 ± 0.14^bc^	26.47	1.88 ± 0.11^b^	25.09	2.00 ± 0.14^b^	24.81
Nisin + Gingerol 1.5%	2.00 ± 0.10^d^	52.94	1.25 ± 0.14^cd^	48.77	1.22 ± 0.12^de^	55.15	1.34 ± 0.16^cd^	46.61	1.55 ± 0.10^c^	41.73
Nisin + Gingerol 2.5%	1.50 ± 0.08^e^	64.71	1.00 ± 0.10^d^	59.02	1.00 ± 0.10^e^	63.24	1.00 ± 0.12^e^	60.16	1.00 ± 0.12^d^	62.41

Note

Means in the same column carrying different superscript letters are significantly different at *p* < .05, *n* = 5. Reduction % = Control−After treatment/Control × 100.

Abbreviations: MPN, most probable number of coliforms; TEC, total enterobacteriaceae count; TMC, total mold count; TPC, total plate count; TPsC, psychrophilic.

**Table 2:** Ameliorative effects of nisin, gingerol, and a mixture of both on the production of biogenic amines in the camel meat

	Cadaverine	Putrescine	Spermine	Tyramine	Total BA
Control	0.25 ± 0.05^a^	0.45 ± 0.08^a^	0.11 ± 0.13^a^	0.22 ± 0.04^a^	1.03 ± 0.11^a^
Nisin 0.5%	0.15 ± 0.04^b^	0.31 ± 0.04^b^	0.08 ± 0.15^ab^	0.14 ± 0.02^b^	0.68 ± 0.09^b^
Nisin 1.5%	0.11 ± 0.03^bcd^	0.22 ± 0.02^cd^	BDT	0.09 ± 0.01b^c^	0.42 ± 0.05^c^
Nisin 2.5%	0.05 ± 0.01^ef^	0.11 ± 0.001^e^	BDT	0.02 ± 0.001^e^	0.18 ± 0.01^e^
Gingerol 0.5%	0.12 ± 0.03^bc^	0.27 ± 0.07^bc^	0.05 ± 0.01^b^	0.11 ± 0.01^bc^	0.55 ± 0.07^bc^
Gingerol 1.5%	0.07 ± 0.01^de^	0.15 ± 0.03^de^	BDT	0.05 ± 0.01^d^	0.27 ± 0.05^d^
Gingerol 2.5%	0.02 ± 0.001^f^	0.07 ± 0.02^e^	BDT	BDT	0.09 ± 0.001^f^
Nisin + Gingerol 0.5%	0.09 ± 0.02^cd^	0.21 ± 0.06^cd^	0.02 ± 0.001^c^	0.10 ± 0.01^c^	0.42 ± 0.01^c^
Nisin + Gingerol 1.5%	0.05 ± 0.01^d^	0.11 ± 0.01^e^	BDT	0.03 ± 0.001^de^	0.19 ± 0.01^e^
Nisin + Gingerol 2.5%	BDT	0.03 ± 0.001^f^	BDT	BDT	0.03 ± 0.001^g^

Note

Means in the same column carrying different superscript letters are significantly different at *p* < .05, *n* = 5.

Abbreviations: BDT, below detection limits; TBA, Total biogenic amines.

## 4 CONCLUSIONS

In conclusion, the results of this study indicated unsatisfactory hygienic measures adopted during the handling of camel meat and edible offal in the slaughterhouses and local markets in Egypt, leading to poor meat quality of the retailed products. Therefore, strict hygienic practices should be followed during the handling of camel meat and offal. Furthermore, using nisin, gingerol, or preferably a combination of both is a promising tool to reduce the microbial load of the camel meat and edible offal.

## CONFLICT OF INTEREST

The authors declare that they do not have any conflict of interest.

## ETHICAL APPROVAL

This study does not involve any human or animal testing.

## INFORMED CONSENT

Written informed consent was obtained from all study participants.
